# Soft mobile robot inspired by animal-like running motion

**DOI:** 10.1038/s41598-019-51308-4

**Published:** 2019-10-11

**Authors:** Tongil Park, Youngsu Cha

**Affiliations:** Center for Intelligent & Interactive Robotics, KIST, Seongbuk-gu, Seoul 02792 Republic of Korea

**Keywords:** Mechanical engineering, Applied physics

## Abstract

There is a considerable demand for legged robots with exploring capabilities such as passing through narrow pathways. Soft robots can provide a solution for such applications. Here, we propose a soft legged mobile robot with bimorph piezoelectric main body and pre-curved piezoelectric legs. We experimentally demonstrate the performance of the soft mobile robot. The mobile robot can move 70% of the body length per second. In addition, we investigate physical mechanisms behind the locomotion of the mobile robot using a numerical simulation. Interestingly, the mobile robot generates an animal-like running motion. We find that the amplitude difference of the legs, depending on the leg activation condition, may affect the performance of the robot. We also confirm that the soft mobile robot can maintain the movement under impulsive shock owing to its flexibility.

## Introduction

Legged robots have received a lot of attention in the past decades owing to the compelling characteristics of exploring challenging artificial and wild terrains^1–12^. These robots are used in various environments including hazardous situations and urban search and rescue work^13,14^.

Inspired by walking and running motions of animals and insects, many researchers have proposed biomimetic legged robots that can achieve terrestrial mobility with legs^2,5,8,10,11^. These robots have the advantages of stability and performance using findings in animal-like walking and running motions^1–3,8,15^. Most developed legged robots are composed of powerful motors or hydraulic actuators and can operate under rugged outdoor conditions with their dynamic mobility^2,8,11^. Although many problems related to limited external ground, environmental sensing, control, and planning remain, quadruped robots have abundant exploration capabilities^2,8,11,16^.

As well as large-scale legged robots, there is considerable need for small-scale robots to explore narrow passages and holes by using novel classes of actuators based on new materials, such as electro-active materials^5,10,17–23^. These small-scale legged robots can be divided into two types: i) a robot with legs activated by embedded actuators and ii) a robot with legs passively moved by body actuation. Specifically, the former directly actuates its legs to generate walking motion^5,10,20,21,23,24^, whereas the latter has passive legs attached to the actuated body. The traveling wave is generated by the movement of the body structure, resulting in the locomotion^17,18,22^. These robots typically utilize piezoelectric beam oscillation.

In the development of small-scale legged robots, soft robots have been reported^25–27^. Soft robotics can have a lot of benefits, such as body transformation and shock absorption^28–31^. These characteristics are essential and attractive features for mobile robots exploring unpredictable situations and environments.

**Figure 1:**
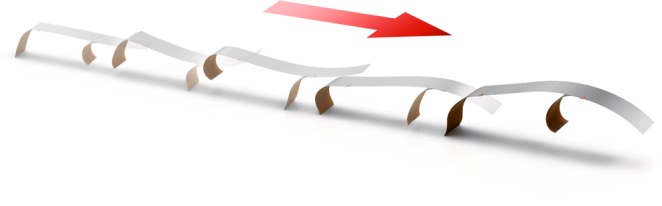
Overlapped images of the mobile robot. The robot consists of the main body and two legs. The materials of the robot are flexible and electrically activated.

In this study, we introduce a soft mobile robot based on a flexible piezoelectric film as shown in Fig. 1. Notably, the robot has a flat main body and two pre-curved legs. This mobile robot achieves high resilience because its body consists of soft materials and also, takes the form of a legged robot. Inspired by the motion of quadruped vertebrates, it is designed to exploit not only the postures of the main body, but also the relative motion of the front and hind legs. We experimentally demonstrate the characteristics of the robot. In particular, by changing the leg condition, we examine noticeable changes in mobility. In addition, we describe the physical mechanisms of the soft mobile robot by a numerical simulation and reveal the relation between the mobility of the robot and the movement of the legs.

## Results

### Fabrication procedure

**Figure 2:**
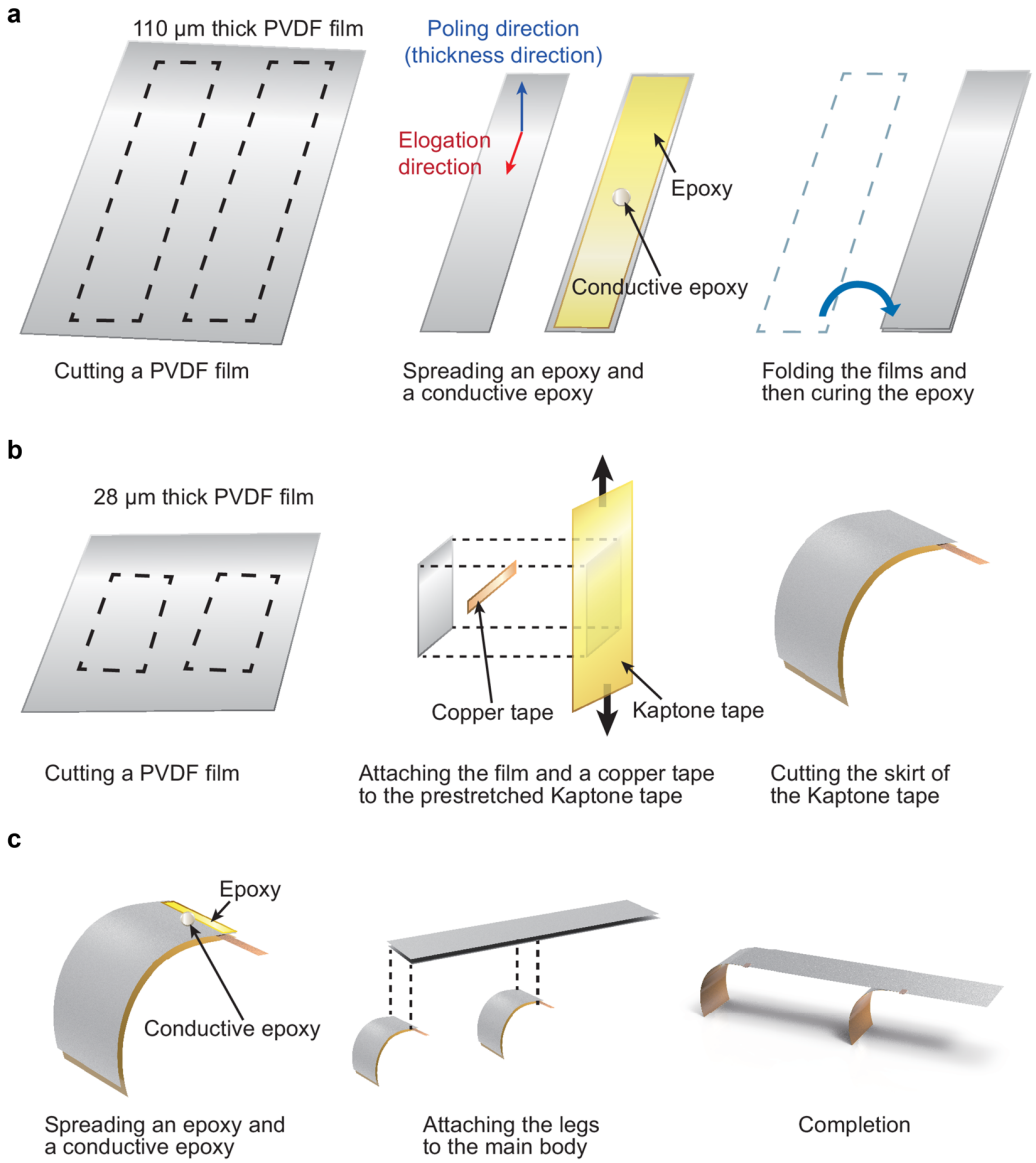
Fabrication procedure. (**a**) Fabrication of the main body. (**b**) Fabrication of the legs. (**c**) Combining the main body and the legs.

The flexible mobile robot was composed of two major parts: the main body and legs. To achieve good resilience, we fabricated both parts using a polyvinylidene fluoride (PVDF) film, which is highly flexible and is actuated by an electric input. The length and width of the main body were 50 mm and 10 mm, respectively. The legs had the same width as the body, and their length was 20 mm. We fabricated the main body as a bimorph PVDF actuator. As shown in Fig. 2a, a 110 *μ*m-thick PVDF film was cut in two prescribed rectangular shapes. To connect both films electrically, we covered a small portion of the film with a conductive epoxy (Epoxy Adhesive 8331, M.G. Chemicals Ltd.), and an epoxy (Epoxy Adhesive DP460, 3M Co.) was covered on the remaining area of the film. We arranged the PVDF films in series, which refers to the case where the poling directions of the films are opposite (Fig. S1). To be specific, a deformation in the elongation direction was generated by the response to a voltage differential across the PVDF film in the poling direction. When a voltage applied to the film composite, one of the PVDF films elongates while the other shrinks since the PVDF films were connected in series (Fig. S1). We attached the films and cured the epoxy for 24 h at 20 °C.

For legs, we fabricated pre-curved unimorph actuators using a 28 *μ*m-thick PVDF film^23^. The film was cut in a rectangular pattern (Fig. 2b). For the pre-curved structure, we stretched a polyimide tape (8997, 3M Co.) with a strain rate of 0.01. We then attached the PVDF film and a piece of copper tapes to the pre-stretched tape and the remained skirt of the tape was removed. The curvature of the pre-curved legs was about 115 m^−1^.

As depicted in Fig. 2c, the legs adhered to the main body. We selected the locations of both legs where the movement of the main body was large enough to effectively transmit the motion of the main body to the legs and the robot was able to maintain stable position. In this manner, we placed the front legs around the center of the main body and the hind legs at the end of the main body; both positions were out of the node of mode shapes where the parts of a beam seldom move. In addition, the front leg was slightly moved to the front of the main body from the center of it since the robot was unstable when the front leg was at the exact center of the main body. The exact locations of the front leg and the hind leg were 15 mm and 46 mm from the head of the robot, respectively. To connect the legs to the main body electrically, we utilized a folded conductive copper tape between the leg bottom and the body top surfaces and a conductive epoxy between the leg top and the body bottom surfaces. We attached the front leg and the hind leg in opposite polarity (Fig. S1). The upper film of the main body and the hind leg had the same polarity, and the bottom film of the main body and the front leg had the same polarity (Fig. S1). Quadruped vertebrates move forward utilizing their bodies as well as legs. Specifically, we observed that in running quadruped animals, the front and hind legs move in the opposite directions^32^. The hind legs are stretched when the hind feet strike the ground, and the forelimbs are pulled to prepare to strike the ground. Our robot mimicked the running motion of quadruped animals. The completed soft mobile robot is extremely lightweight (0.32 g).

### Mobility of the soft mobile robot

We experimentally investigated the mobility of the proposed soft mobile robot. We applied a square wave voltage signal to the robot. The amplitude of the applied voltage was peak-to-peak 130 V (DC 65 V ± AC 65 V). The PVDF film is quite thin. High voltage inputs over critical level could cause electrical short-circuit at the edge of the film and inflict serious damages on the PVDF components. For this reason, we chose the square wave with DC 65 V ± AC 65 V as the input driving signal for safe and maximal operation of the proposed mobile robot.

We conducted preliminary experiments to find the frequency at which the mobile robot performs best. The experiments were performed in the range of 1 Hz–200 Hz. The robot presented optically detectable movement only at around 57 Hz and 160 Hz. In other words, driving input signals except both frequencies did not generate meaningful movement. We select the testing frequency of 160 Hz wherein the robot has the highest velocity.

We tried to find the reasons why the robot has confined frequency ranges by inquiring into the motion of the robot at the frequencies. However, since the displacement of the robot is of microscopic scales and the components of the robot is highly soft, it is hard to find a proper method that is able to determine the motion of flexible robots. For this limitation, we tried to analyze the motion of the robot utilizing experimental ways. We focused on the main body of the robot and tried to figure out the state of the main body at the frequency of 57 Hz and 160 Hz. Furthermore, owing to a lot of obstacles or the significant level of sophistication to construct a theoretical model with a pre-curved, pre-stretched, and layered structure, the tasks did not involve the front and hind legs of the robot.

To analyze the main body, we simplified it as a composite beam. We compared the resonance frequency under the free-free condition based on the weighted frequency to the frequency at which the robot presented noticeable movement^33^. First, we conducted an experiment to find the resonance frequency of the beam under fixed-free condition. To be specific, we clamped the short side of the beam at a stand depicted in Fig. S2. The displacement at the end of the main body was recorded according to the frequencies of input signals (Fig. S3). Then, by utilizing the obtained resonance frequency and the equation related to the weighted frequency under fixed-free condition, we obtained combination of the unknown properties.

With the value combined with unknown properties and the equation of the weighted frequency under the free-free condition, we determined the resonance frequency of the main body without legs under the free-free condition. From the calculation, we obtained the the frequency of 147 Hz. Despite of the small difference with the experimental finding of 160 Hz, the calculated value indicates that the large movement of the main body at the resonance frequency is a factor leading the best mobility of the robot. Incidentally, the discrepancy between the obtained value and the experimental results may be linked to the influence of robot’s surroundings, such as the existence of legs, gravity, and a floor. Specifically, the existence of legs and gravity seems to affect the mass distribution and the partial stiffness. In addition, it could be speculated that a floor beneath the robot influences on its boundary condition.

**Figure 3:**
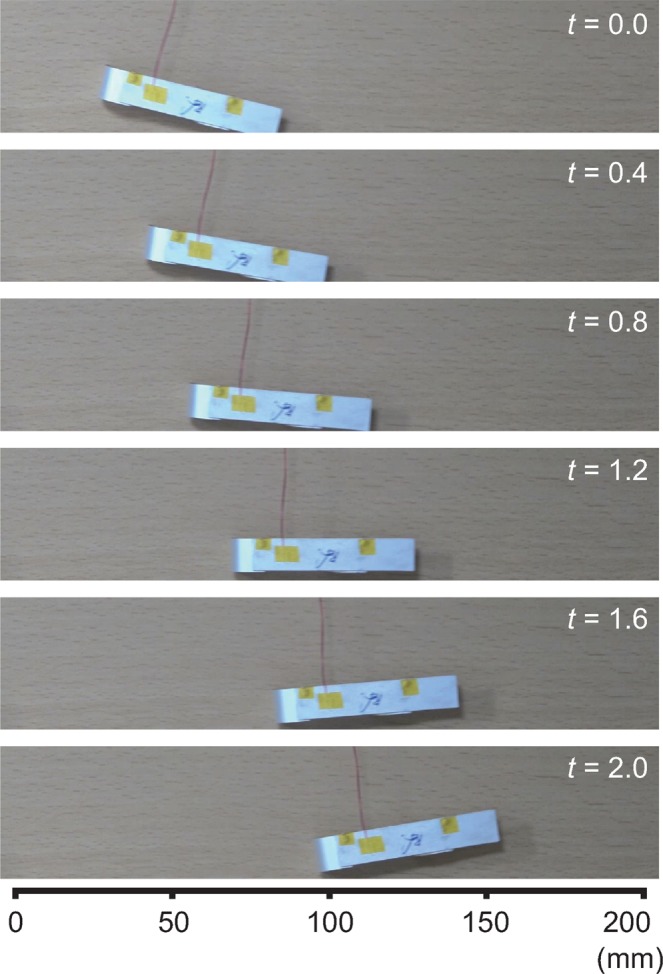
Images of the mobile robot. All legs of the robot are activated. The driving voltage signal is based on the square wave of 160 Hz.

**Figure 4:**
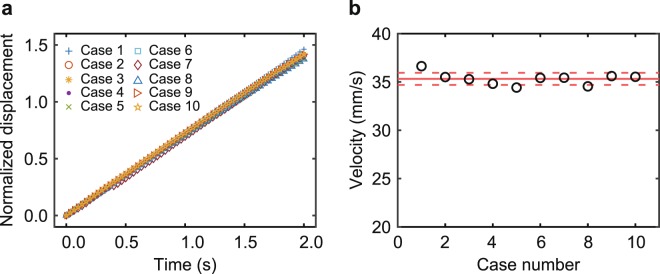
Normalized displacement over time and velocity of the robot. (**a**) Normalized displacement of the robot over time for ten cases. (**b**) Velocity of the robot and its standard deviation for ten cases. The circles are the velocity of each case. The solid red line is the mean velocity, and the red dashed lines are the standard deviation of the velocity relative to the mean velocity.

Figure 3 shows the displacement of the soft mobile robot for 2 s, where both legs are in operation. We can observe that the robot moves 70 mm from left to right. The robot movement is not a direct line because of the tension of power source wires. We repeated ten experiments for each case since the movement of the robot was affected by connecting wires. From the results, the average displacement and velocity of the robot were obtained. Figure 4a shows the displacement of the robot in the lateral direction for ten cases. The value is normalized by the length of the main body (*L*). In all cases, the normalized displacement increases with time, with minor discrepancies. The time taken for the robot to move as much as its body length was approximately 1.4 s.

The velocity data of the robot are shown in Fig. 4b, where circles are the velocity for each case, a solid red line is the mean velocity over ten runs, and dashed lines are the standard deviation of the velocity. The average velocity of the proposed mobile robot (n = 10) is 35.3 mm/s. The velocity value indicates that the robot can move over 70% of its body length per seconds.

To investigate the effect of the front and hind legs, we performed experiments under four different leg conditions: both legs activated, only front leg activated, only hind leg activated, and both legs deactivated. For all leg conditions, the main body is always actuated. A leg can be deactivated by detaching the copper tape, which provides the electrical connection between the main body and the leg. For each case, we also repeated the experiments 10 times and calculated the mean velocity and the standard deviation over ten runs.

**Figure 5:**
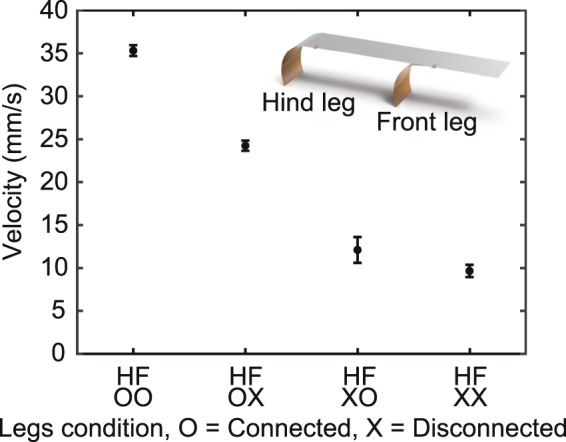
Mean velocity and standard deviation for four different leg conditions. Averaged velocity and corresponding standard deviation for each leg conditions. ‘F’ represents the front leg, and ‘H’ represents the hind leg.

Figure 5 shows the velocity for the four different leg conditions. Compared to other leg conditions, the robot with both legs electrically activated shows superior performance of velocity, as seen in Fig. 5. When both legs are activated, the velocity of the robot increases by 3.7 times, as compared to the case when both legs do not work. In the previous work, we confirmed that the movement of the main body makes a great impact on the performance of mobility. With the results of the experiments, it appears that the large movement of the main body at the resonance frequency builds foundations of mobility of the robot and its legs amplifies the mobility.

When one of the two legs does not work, we also observe a significant velocity drop. Interestingly, the velocity drop as the hind leg is deactivated is considerably greater than that when the front leg is deactivated. When the front leg is electrically disconnected, the velocity reduces to 69.8% of that when both legs are connected. Meanwhile, when the hind leg is electrically disconnected, the velocity decreases to 34.3% of that when both legs are activated. These results demonstrate that the hind leg has a better ability to improve the mobility of the robot than the front leg. Since the components of legs and the input condition on legs are equal, the legs have almost identical displacement. However, when boundary conditions become different, it could suppress the movement of legs. To be specific, owing to the center of gravity, the larger mass could be concentrated on the front leg, leading the movement of the front leg to be suppressed.

### Physical mechanisms of the soft mobile robot

The soft mobile robot has several noticeable tendencies in mobility under different leg condition. However, it is difficult to clearly understand the physical mechanisms responsible for these trends from the optical measurement, because the robot is operated by the microscopic displacement of piezoelectric actuators. To understand the physical mechanisms behind the flexible mobile robot, we conducted a numerical simulation using COMSOL Multiphysics 5.3a. In particular, we performed the simulation in two-dimensional space and utilized piezoelectric device module/physics in the model. To apply the stretched condition to the polyimide tape of the legs, we applied stress to the end of the legs, resulting in a curvature in the legs. Then, we applied harmonic excitation to the structure at a frequency of 160 Hz. The boundary condition of the structure is the free-free condition. That is, the simulation model did not include floors beneath the robot and the effect of gravity due to the complex non-linearity from irregular contact between the legs of the robot and the floor.

We verified the computational model for adequate simulation. We conducted the simulation of beams under the fixed-free condition, which is identical to the experiment in the previous section. We extracted the reliable thickness of the epoxy between the PVDF films of the main body by comparing frequency values from the simulation and the experiment in the last section. Through the obtained thickness, we were able to achieve the reliable results of simulations.

**Figure 6:**
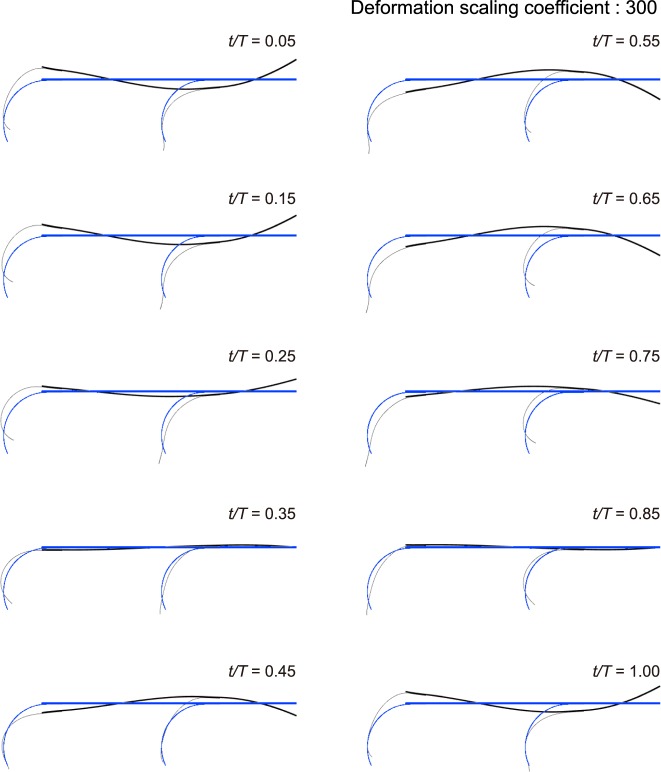
Locomotion gait of the mobile robot at 160 Hz. The mobile robot is in the case where both legs are electrically activated. A blue lines is the initial position, and a black line is the position at the given time.

Figure 6 illustrates the time sequence motion of the robot when all legs are electrically connected. The time (*t*) is normalized by dividing the period of the input driving signal (*T*). When the normalized time (*t*/*T*) is 0.05, the body has a U shape, and the location of the front leg is below that of the hind leg. As the normalized time is increased until *t*/*T* = 0.35, the front leg moves upward and the hind leg moves downward. Simultaneously, the front leg extends, generating a motion which is analogous to striking to the ground. Unlike the front leg, the hind leg is flexed, which resembles the movement of leaving the ground. We noted that the front and hind legs move in opposite directions when both legs are electrically stimulated. After *t*/*T* = 0.35, the structure of the body starts to vary from the U shape to the inverse shape, carrying the front leg upward and the hind leg downward. Concurrently, the hind leg extends, showing hind leg footfall. Meanwhile, we can see the shrunk front leg. Majority of quadruped vertebrates utilize both their back and legs to achieve considerable speeds^32,34^. We observed that the robot inspired by running animals also employs both the postures of the body and the relative movement of the legs to obtain its mobility.

Additionally, when one of the legs is electrically deactivated, we can observe that the inactive leg experiences a considerable reduction in the movement in the lateral direction as shown in Figs S4–S6.

**Figure 7:**
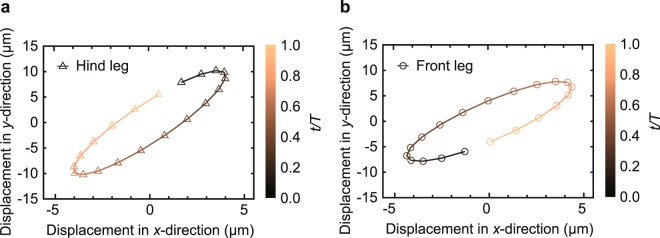
Displacement of the hind and font leg. (**a**) Trajectory of the hind leg. (**b**) Trajectory of the front leg.

To monitor the movement of the legs specifically, the displacement of the end point of the legs was traced in the horizontal (*x*) and vertical (*y*) direction for one stride as shown in Fig. 7a,b. Both legs move in an elliptical shape and the clockwise direction. Meanwhile, there is a significant distinction in the moving direction between the hind leg and the front leg. The hind leg is initially located at the upper right corner (Fig. 7a), whereas the front leg starts to move at the bottom left corner (Fig. 7b). Specifically, the hind legs go diagonally downward when the front leg moves diagonally upward, and vice versa. By assuming the ground underneath the robot, we can imagine that the hind leg strikes the ground when the front leg leaves the ground, and vice versa.

When the robot has an inactive leg, the lateral displacement of the inactive leg is reduced up to 49% of that of the active leg (Fig. S7). In addition, when a leg is inactive, its end shows counterclockwise movements. It shows that these reductions of the lateral displacement of the leg and its counterclockwise motion could negatively affect the performance of the robot. Compared to the robot without any active leg, the sum of the front and hind leg displacements is 200% larger. This tendency corresponds to the experimental velocity result. It is reasonable to suppose that the motion of the main body enables the robot to move, and the movement of the legs promotes the performance of the robot.

### Ability to move under impulsive shock

**Figure 8:**
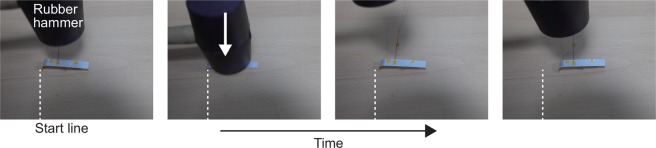
Verifying the capability to operate under impaction. During the locomotion, we apply impacts to the mobile robot using a rubber hammer. It shows that the robot can keep moving after the impacts from the hammer.

Since extant mobile robots have been required to explore territories with unknown surroundings wherein a sudden external impact, such as falling rocks, may occur, the ability to rebound from being accidentally squashed after pressing needs for mobile robots. We conducted additional experiments to confirm that the mobile robot maintains its initial shape and performance of mobility after an external shock. As shown in Fig. 8, we used a rubber hammer to examine and test the shape and mobility of the robot after a shock. Finally, we observed that it can keep moving after an impulsive shock. The impact from the hammer cannot inflict serious damage on the elements of the robot, and thus it can be restored to its original shape and continue to operate because all the components of the robot were made up of soft and flexible materials. For the above results, we confirm that the mobile robot suggested in this paper can be utilize for travelling unknown surroundings with the possibility of an impact.

## Discussion

We proposed a mobile robot here that has not only considerable mobility, but also excellent resilience. We improved the performance of the robot by fabricating pre-curved legs using a combination of a PVDF sheet and a prestretched polyimide tape. Inspired by running quadruped animals, we fabricated the robot to generate the postures of its body and the relative motion of its legs. The mobility of the robot was investigated experimentally for several conditions of the legs. The mobile robot can travel at 70% of its body length a second. We found that the mobility of the robot is altered critically depending on whether the legs are operated or not.

Moreover, using a numerical simulation, we studied the physical mechanisms of the robots that are difficult to observe experimentally. From the results of the simulation, we discovered that the movement of the robot is analogous to running quadruped animals with respect to the fact that both exploit the movement of their body and legs. We confirmed that the active leg shows a similar motion of striking the ground, which results in improving the mobility performance of the robot.

The proposed soft robot can be utilized in exploring environments that are challenging for a person to enter because a sudden and an unknown impact may occur. Because all the elements of the robot including the main body are made of flexible materials, the robot can be operated after an impulsive impact on the robot.

As a fundamental step of the novel type of soft mobile robots inspired by an animal running motion, we focused on finding its unique characteristics and tendencies by utilizing experiments and computational simulation. The interaction between the robot’s legs and the floor beneath the robot is so complicated that detailed physical mechanisms underlying their effects on the mobility still remain unclear. Thus, in the future, the instantaneous motion of the robot relative to the floor and the development of a state of the robot should be considered in order to fully understand the mobile performance of a soft mobile robot. Additionally, the various parameters, such as the geometry of the robot and the kinetic of the motion, should also be explored in future work.

## Methods

To apply DC 65 V ± AC 65 V to the robot, we utilized a voltage amplifier (PA95, Apex Microtechnology Co., Ltd.) and an evaluation kit (EVAL 23, REVB, Apex Microtechnology Co., Ltd.), where the gain of the amplifier is 100 (Fig. S8). Power supply (MK-1000CK, MK Power) was connected to the amplifier to provide an operation voltage. The operation voltage was DC 180 V. A waveform generator (33500B, Keysight Technologies) provided the amplifier with the control signal. In addition, the main body of the mobile robot was wired to the amplifier. We utilized a PVC plate as a boundary ground. A camera (DSC-RX10M3, Sony Co.) was utilized to record the optical data. The camera was installed above the robot and recorded video at a rate of 120 frames per second. The velocity of the robot was evaluated using the obtained optical data. We tracked a trace marker on the robot using a color tracking code (Matlab, Mathwork).

## Supplementary information

Mobility of the soft mobile robot

Locomotion gait of the mobile robot

Impact test of the mobile robot

Supplementary figures
