# Supplementary material for: Soft mobile robot inspired by animal-like running motion
Source: Sci Rep. 2019 Oct 11;9:14700. doi: 10.1038/s41598-019-51308-4 (PMC6788992; doi:10.1038/s41598-019-51308-4)
Supplement: Supplementary file 4 — Supplementary figures [file 41598_2019_51308_MOESM4_ESM.pdf]

Soft mobile robot inspired by animal-like running motion

Tongil Park[1] **and Youngsu Cha**[1*]

[1]Center for Intelligent & Interactive Robotics, KIST, Seongbuk-gu, Seoul, 02792, Republic of Korea
[*]Corresponding author, givemong@kist.re.kr

ABSTRACT

There is a considerable demand for legged robots with exploring capabilities such as passing through narrow pathways. Soft robots can provide a solution for such applications. Here, we propose a soft legged mobile robot with bimorph piezoelectric main body and pre-curved piezoelectric legs. We experimentally demonstrate the performance of the soft mobile robot. The mobile robot can move 70 % of the body length per second. In addition, we investigate physical mechanisms behind the locomotion of the mobile robot using a numerical simulation. Interestingly, the mobile robot generates an animal-like running motion. We find that the amplitude difference of the legs, depending on the leg activation condition, may affect the performance of the robot. We also confirm that the soft mobile robot can maintain the movement under impulsive shock owing to its flexibility.

Supplementary materials

Figure S1. Poling directions of the PVDF films of the robot.
Figure S2. Experimental setup for measuring the displacement of the bimorph PVDF beam in fixed-free condition.
Figure S3. Results of natural frequency experiments of a PVDF composite beam.
Figure S4. Locomotion gait of the mobile robot at 160 Hz (only hind leg activation).
Figure S5. Locomotion gait of the mobile robot at 160 Hz (only front leg activation).
Figure S6. Locomotion gait of the mobile robot at 160 Hz (both legs deactivation).
Figure S7. Displacement of the hind and font leg.
Figure S8. Experimental setup for measuring the movement of the mobile robot.

Movie S1. Mobility of the soft mobile robot.
Movie S2. Locomotion gait of the mobile robot.
Movie S3. Impact test of the mobile robot.

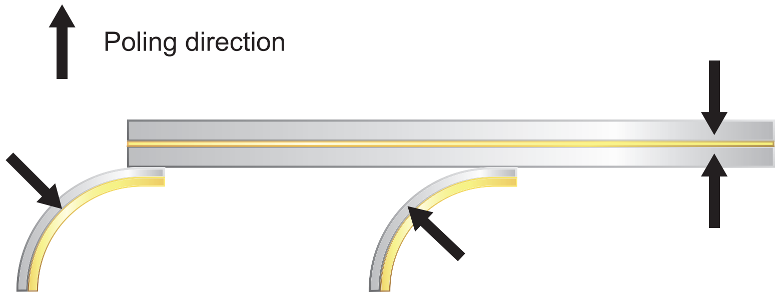

Figure S1. Poling directions of the PVDF films of the robot The PVDF films of the main body was placed in series, and the poling directions of the legs are opposite.

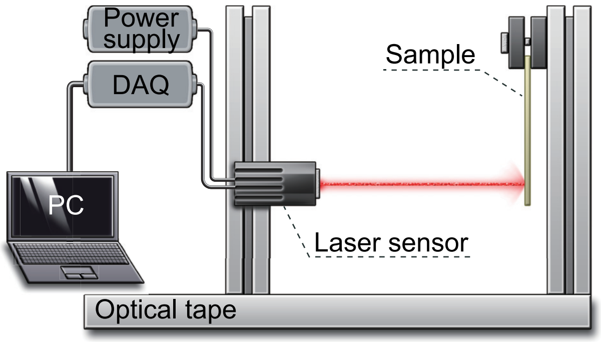

Figure S2. Experimental setup for measuring the displacement of the bimorph PVDF beam in fixed-free condition The displacement of the bimorph PVDF structure was measured by lase displacement sensor.

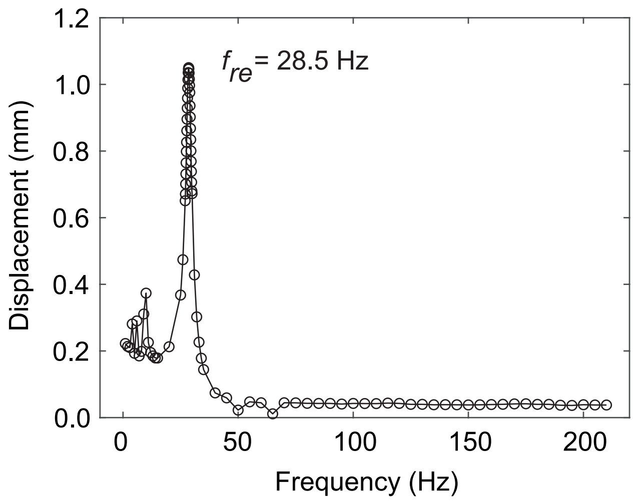

Figure S3. Results of natural frequency experiments of a PVDF composite beam The displacement at the end of the PVDF composite beam was measured according to the frequencies of the input signal. The results showed that the natural frequency of the beam was 28.5 Hz.

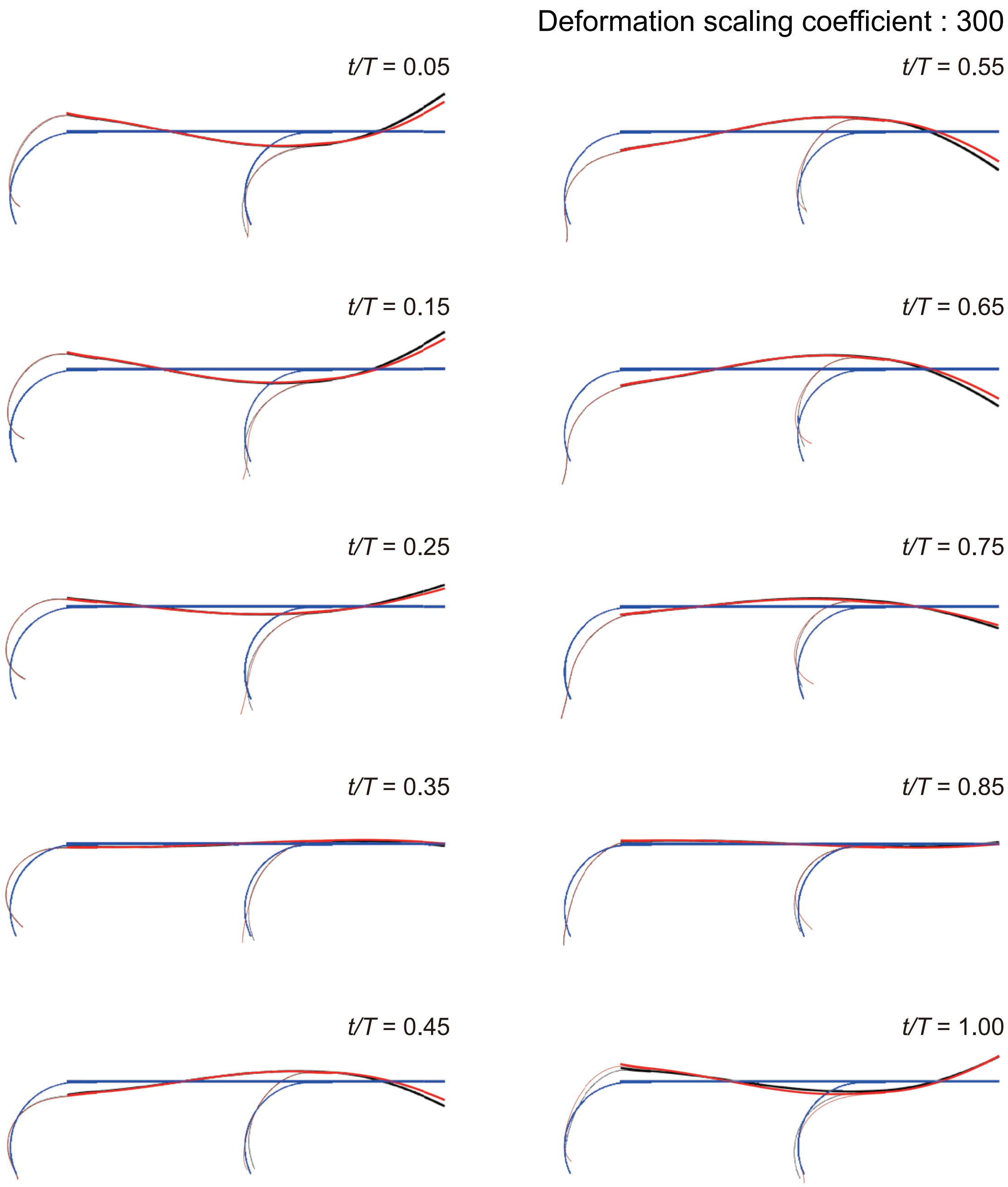

Figure S4. Locomotion gait of the mobile robot at 160 Hz (only hind leg activation). The mobile robot is in the case where only hind leg is electrically activated. A blue line is the initial position, a black line is the position at the given time, and a red line is the position as all of the legs is electrically activated.

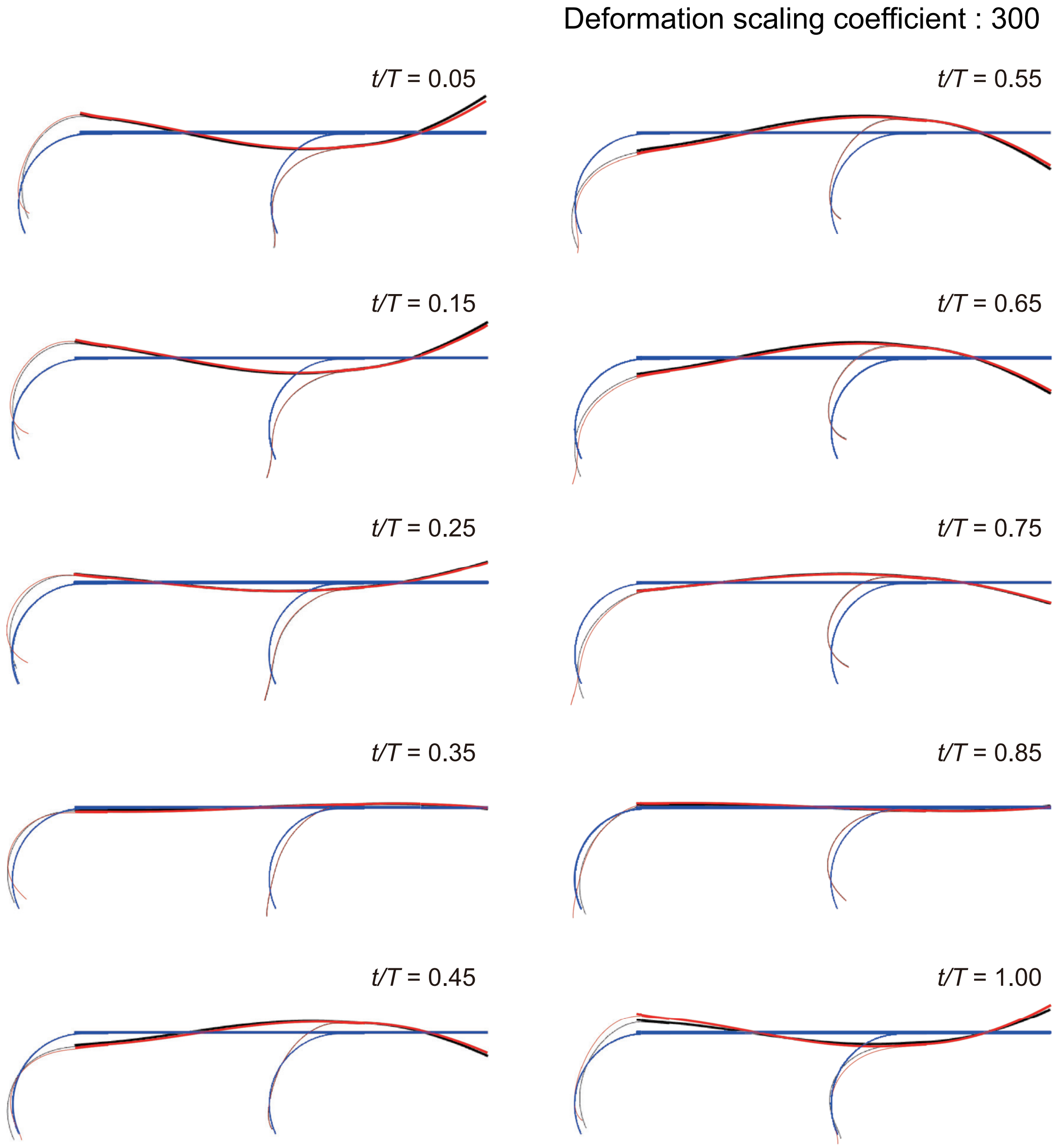

Figure S5. Locomotion gait of the mobile robot at 160 Hz (only front leg activation). The mobile robot is in the case where only font leg is electrically activated. A blue line is the initial position, a black line is the position at the given time, and a red line is the position as all of the legs is electrically activated.

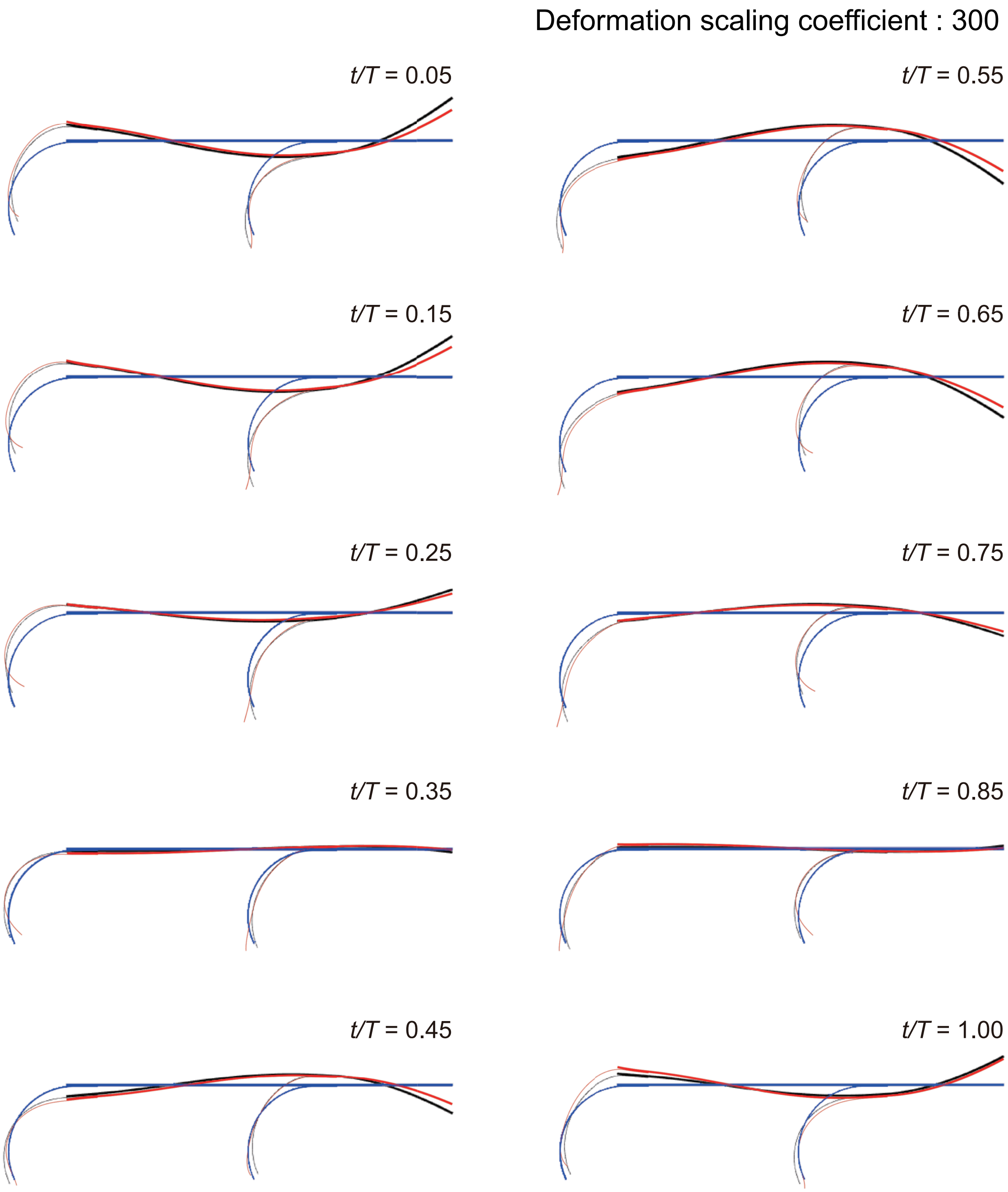

Figure S6. Locomotion gait of the mobile robot at 160 Hz (both legs deactivation). The mobile robot is in the case where both legs are electrically deactivated. A blue line is the initial position, a black line is the position at the given time, and a red line is the position as all of the legs is electrically activated.

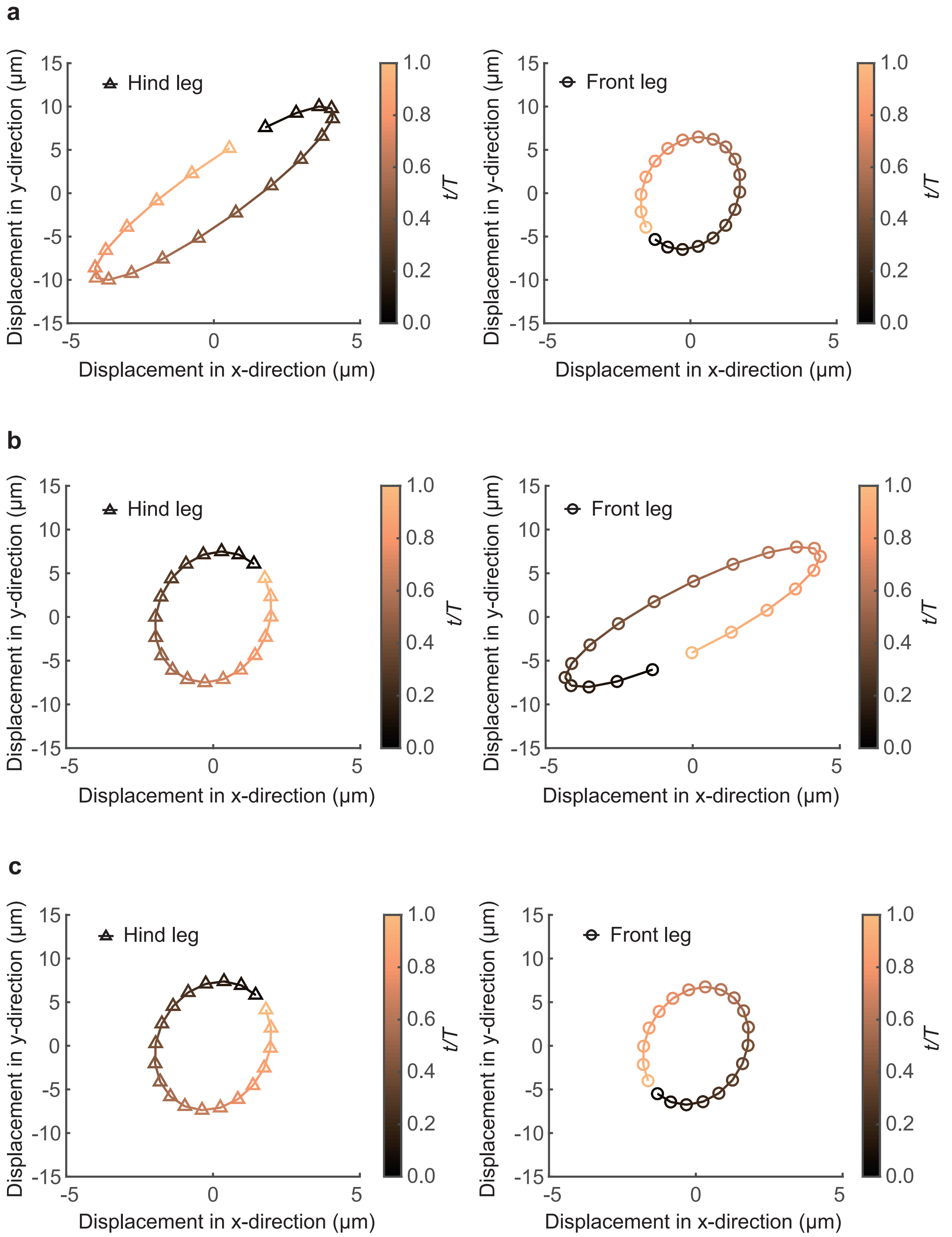

Figure S7. Displacement of the hind and font leg. (**a**) The hind leg is activated, and the front leg is deactivated. (**b**) The hind leg is deactivated, and the front leg is activated. (**c**) Both legs are deactivated

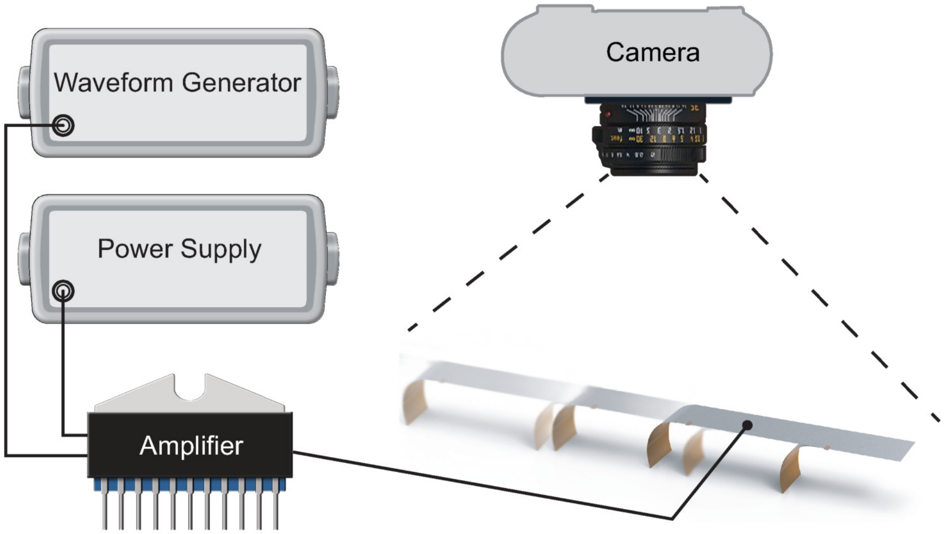

Figure S8. Experimental setup for measuring the movement of the mobile robot The driving signal was applied to the mobile robot utilizing an amplifier, a power supply, and an waveform generator. A camera recorded the movement of the robot.

Movie S1. Mobility of the soft mobile robot. The movie shows the mobility of the robot when both legs are activated. The driving voltage signal is square wave of 160 Hz.

Movie S2. Locomotion gait of the mobile robot. The movie shows the motion of the soft mobile robot obtained by numerical simulation. In the simulation, all the legs are in operation.

Movie S3. Impact test of the mobile robot. During operating of the soft mobile robot, we apply the several impact to the robot using a rubber hammer. It shows that the robot can continue operation after several impacts from the hammer.
